# Effects of HSP90 inhibitor 17-allylamino-17-demethoxygeldanamycin (17-AAG) on NEU/HER2 overexpressing mammary tumours in MMTV-NEU-NT mice monitored by Magnetic Resonance Spectroscopy

**DOI:** 10.1186/1756-0500-5-250

**Published:** 2012-05-23

**Authors:** Loreta M Rodrigues, Yuen-Li Chung, Nada M S Al Saffar, Swee Y Sharp, Laura E Jackson, Udai Banerji, Marion Stubbs, Martin O Leach, John R Griffiths, Paul Workman

**Affiliations:** 1 Cancer Research UK Cambridge Research Institute, Li Ka Shing Centre, Robinson Way, Cambridge, CB2 0RE, UK; 2 Cancer Research UK and EPSRC Cancer Imaging Centre, The Institute of Cancer Research and The Royal Marsden NHS Foundation Trust, Sutton, Surrey, SM2 5PT, UK; 3 Cancer Research UK Centre for Cancer Therapeutics, The Institute for Cancer Research, Sutton, Surrey, SM2 5NG, UK

**Keywords:** Magnetic resonance spectroscopy, MMTV-NEU-NT tumours, ERBB2/ HER2/ NEU, HSP90, 17-AAG

## Abstract

**Background:**

The importance of ERBB2/NEU/HER2 in the response of breast tumours to the heat shock protein 90 (HSP90) inhibitor 17-allylamino-17-demethoxygeldanamycin (17-AAG; tanespimycin) has been demonstrated in the clinic. ERBB2 is an oncoprotein client that is highly dependent on HSP90. This and other oncogenic client proteins (e.g. B-RAF, C-RAF, ALK and CDK4) are depleted by 17-AAG in both animal tumours and patients. Here we investigate by Magnetic Resonance Spectroscopy (MRS) the metabolic response of 17-AAG in spontaneous, NEU/HER2 driven mammary tumours in transgenic MMTV-NEU-NT mice and in cells isolated and cultured from these tumours.

**Methods:**

Mammary tumours were monitored by ^31^P MRS *in vivo* and in tumour extracts, comparing control and 17-AAG treated mice. A cell line derived from NEU/HER2 mammary tumours was also cultured and the effect of 17-AAG was measured by ^31^P MRS in cell extracts. Molecular biomarkers were assessed by immunoblotting in extracts from cells and tumours. For comparison of tumour volume, metabolite concentrations and Western blot band intensities, two-tailed unpaired t-tests were used.

**Results:**

The NEU/HER2 mammary tumours were very sensitive to 17-AAG and responded in a dose-dependent manner to 3 daily doses of 20, 40 and 80mg/kg of 17-AAG, all of which caused significant regression. At the higher doses, ^31^P MRS of tumour extracts showed significant decreases in phosphocholine (PC) and phosphoethanolamine (PE) whereas no significant changes were seen at the 20mg/kg dose. Extracts of isolated cells cultured from the mammary carcinomas showed a significant decrease in viable cell number and total PME after 17-AAG treatment. Western blots confirmed the expected action of 17-AAG in inducing HSP72 and significantly depleting HSP90 client proteins, including NEU/HER2 both in tumours and in isolated cells.

**Conclusions:**

The data demonstrate the high degree of sensitivity of this clinically relevant NEU/HER2-driven tumour model to HSP90 inhibition by 17-AAG, consistent with the clinical data, and suggest that the metabolic signature of choline phospholipids obtained by MRS could be useful both as a preclinical and clinical tool for investigating surrogate markers of response to treatment.

## Background

The novel anticancer drug 17-allylamino-17-demethoxygeldanamycin (17-AAG; tanespimycin), which inhibits the molecular chaperone HSP90, has shown activity against breast cancer in the clinic [1-3]. Regressions were seen only in patients with metastatic breast cancer that was positive for ERBB2/HER2, a very sensitive oncoprotein client of HSP90. The ERBB2 receptor tyrosine kinase, a member of the epidermal growth factor receptor (EGFR) family, is overexpressed in 20-30% of primary human breast cancers, and expression correlates with poor patient outcome [4]. ERBB2/HER2 is a target for both antibody-based therapy (as with trastuzumab) and tyrosine kinase-based inhibitors (as with lapatinib), but resistance is seen in the clinic [5]. ERBB2/HER2 is one of the most sensitive client proteins of HSP90 [6,7], and 17-AAG has been shown to cause depletion of ERBB2/HER2 leading to significant growth inhibition in ERBB2/HER2 overexpressing breast cancer cells and tumour xenografts [8], and more importantly to cause regression in trastuzumab-refractory ERBB2/HER2 positive breast cancer patients [1-3]. Data from Phase 2 trials have recently confirmed this impressive anti-tumour activity of 17-AAG and validated HSP90 as a therapeutic target for ERBB2/HER2-driven breast cancer [2].

17-AAG, a derivative of the naturally-occurring ansamycin antibiotic geldanamycin, is known to bind specifically to the ATP site in the NH_2_-terminal domain of the molecular chaperone HSP90 and inhibit its function [7,9]. As well as ERBB2, HSP90 is required for the correct conformation and function of many other oncogenic client proteins, including CDK4, C-RAF, B-RAF, AKT, MET, ALK, EGFR and p53 [10,11].

Several mutant oncoproteins are more dependent than their wild-type forms on HSP90 but ERBB2 is the most sensitive HSP90-dependent client protein identified to date [11,12]. Tumour cells contain HSP90 complexes in a more highly activated, high affinity conformation compared to normal cells, and it has been proposed that this might contribute to malignant progression [13]. HSP90 is therefore a key therapeutic target, since a drug that simultaneously inhibits several oncogenic signaling pathways is likely to be particularly effective [7,11,12,14]. Recent clinical experience indicates that HSP90 inhibitors are showing therapeutic activity in settings where they deplete sensitive HSP90 clients in tumour types that are addicted to those clients, with ERBB2-positive, trastuzumab-refractory breast cancer providing the best example [2,3]. Clinical development of HSP90 inhibitors is focusing heavily on these tumours and additional studies in mechanistically relevant preclinical models are warranted to support and inform the ongoing clinical work.

The development of transgenic mouse models that recapitulate the initial events of ERBB2-induced mammary tumourigenesis has played a key role in understanding the molecular basis of ERBB2-driven tumours [15]. NEU/HER2 is the rat homolog of human ERBB2 and in one transgenic mouse model, MMTV-NEU-NT, that expresses the activated mutant form of NEU under the transcriptional control of the MMTV promoter, the mice develop spontaneous tumours in the mammary gland [16]. This tumour model is particularly suited to studying the effects of 17-AAG since the tumours that arise are driven by the activated form of the NEU/HER2 oncogene and are addicted to it, just as ERBB2 amplified breast cancers in the clinic are driven by and addicted to the amplified ERBB2. The action of 17-AAG on HSP90 promotes the degradation of client proteins through the ubiquitin-proteasome pathway, and such a pleiotropic mechanism may induce complex metabolic effects that may vary from one tumour to another. In the discovery and in early clinical trials of HSP90 inhibitors, it will therefore be important to develop non-invasive monitoring methods that can distinguish these responses. Such methods could then be used in the clinic to select tumours that are likely to respond to treatment, with advantages over more invasive techniques [17-19].

Magnetic resonance spectroscopy (MRS) can be used non-invasively to study the biochemistry and physiology of tumours *in vivo,* and also *ex vivo* on extracts of either tumours or cells. ^31^P MRS has been used to monitor growth and response to therapy both in animal tumour models and in patients [20-24]. Biomarkers for tissue bioenergetics, such as nucleotide triphosphate (NTP), inorganic phosphate (Pi), and intracellular pH (pHi), as well as various phosphorus-containing phospholipid metabolites are readily observed with ^31^P-MRS. The phospholipid metabolites that provide information on membrane metabolism are the phosphomonoesters (PMEs) phosphocholine (PC) and phosphoethanolamine (PE), which are precursors of the phosphatidylcholine and phosphatidylethanolamine in biological membranes, and the phosphodiesters (PDEs) glycerophosphocholine (GPC) and glycerophosphoethanolamine (GPE), which are breakdown products of phosphatidylcholine and phosphatidylethanolamine. The pharmacodynamic actions of many anticancer drugs have been studied, and characteristic ^31^P MRS signatures established (for a review see [25]). Since MRS changes induced by 17-AAG occur in parallel with the expected molecular marker changes, the MRS signatures have the potential to provide surrogate markers of treatment response [26-28]. We therefore set out to look at the MRS signature of the action of 17-AAG in spontaneous, mammary tumours in transgenic MMTV-NEU-NT mice where the tumors that arise are driven by the activated form of the NEU/HER2 oncogene and are addicted to it, similar to ERBB2 amplified breast cancer in the clinic.

## Materials and methods

### Materials

17-AAG for the *in vivo* and *in vitro* study was either provided (dissolved in DMSO at 25mg/mL) by Dr. P. Ivy at the National Cancer Institute (Bethesda, MD) or purchased (powder form) from Alexus Biochemicals (Switzerland) and dissolved in DMSO at 25mg/mL. Dilutions to 2.5mg/mL were made in a vehicle of egg phospholipid supplied by Dr. P. Ivy. Dulbecco’s Modified Eagle Medium (DMEM), penicillin, and streptomycin were purchased from Life Technologies (Paisley, UK) and fetal calf serum (FCS) from PAA labs Ltd. (Somerset, UK). Hypnorm was purchased from Jansen Pharmaceuticals (Buckinghamshire, UK), and Hypnovel was purchased from Roche (Welwyn Garden City, UK). Perchloric acid (PCA) and potassium hydroxide were purchased from Merck (Poole, UK). Sodium 3-trimethylsilyl-2,2,3,3-tetra-deuteropropionate (TSP) was purchased from Goss Scientific Instruments Ltd, UK. All other chemicals were purchased from Sigma (Poole, UK).

### MMTV-NEU-NT tumour model

Female transgenic mice expressing the mutant activated form of rat NEU (NEU-NT) under transcriptional control of the MMTV promoter (MMTV-NEU-NT mice) were purchased from Charles River, UK. The mating strain is the FVB/N mouse and multiple tumours involving the entire mammary epithelium arise synchronously in the mammary gland area [16] between 18-20 weeks of age in 35-50% of the mice.

Mice were maintained under strict inbreeding conditions; health was monitored every 3 months and the presence of the Neu transgene was routinely checked by PCR on tail DNA. Animals were anesthetized with 8mL/kg of a Hypnorm: Hypnovel: water (1:1:2) mixture. All experiments were performed in accordance with the UK Home Office Animals Scientific Procedures Act 1986 and national UK Coordinating Committee on Cancer Research’s (UKCCCR) guidelines [29]. All measures were taken to minimize any pain or discomfort to the animals.

Tumour volume was calculated using the formula (π/6)(d1·d2·d3) where d1, d2 and d3 are the three orthogonal diameters measured by callipers to derive an ellipsoidal volume.

Several cohorts of mice were used: of the treated cohorts, one received 20mg/kg 17-AAG i.p., a second received 40mg/kg and a third received 80mg/kg, once a day for 3 days. The fourth control cohort received vehicle (egg phospholipid in 10% DMSO), once a day for 3 days. *In vivo* MRS was performed on day 0 and day 4, and tumours were freeze-clamped on day 4. Western blot analysis was performed on part of the tumour tissue, and ^31^P and ^1^H MRS were performed on acid extracts of tumour tissue. In a fifth cohort, as part of a regrowth study, the mice received 40mg/kg 17-AAG once a day for 3 days and the tumours were monitored up to 14 days after the end of treatment.

### *In vivo* MRS

*In vivo* magnetic resonance experiments were performed on a Varian Unity Inova 4.7T spectrometer. During the MR experiments, all animals were covered with a warm water blanket and core temperature maintained at 37°C.

For ^31^P MRS, the volume of interest selected from a ^1^H image included as much of the tumour as possible and avoided the underlying body wall. ISIS (image-selected *in vivo* spectroscopy) [30] localized spectra of tumours were acquired using a 10mm, two-turn surface coil, TR of 3s and 240 acquisitions. Spectra were acquired prior to treatment and at day 4 in the cohorts that received 3 doses, at 24 hr in the single dose cohort and at days 4, 8, 11 and 18 in the regrowth cohort. Spectra were quantitated using VARPRO, a time-domain non-linear least squares method [31]. The data were fitted assuming contributions from phosphomonoesters (PME), phosphodiesters (PDE), inorganic phosphate (P_i_), phosphocreatine (PCr), and the α-, β- and γ-nucleoside triphosphate (NTP) resonances.

### High resolution ^1^H and ^31^P MRS of tumour extracts

Part of the freeze-clamped tumour was extracted with 4 volumes of 6% PCA. The neutralized extracts were freeze-dried, reconstituted in deuterium oxide and placed in 5mm NMR tubes. MR spectra were acquired at room temperature on a 500MHz Bruker spectrometer (Bruker Biospin, Coventry, UK).

For ^1^H MRS, the water resonance was suppressed by using gated irradiation centered on the water frequency. TSP (50ul, 5mM) was used for chemical shift calibration and quantitation.

For ^31^P MRS, metal ions were chelated by addition of EDTA (50ul, 60mM) and methylene diphosphonic acid (MDP, 50ul, 5mM) was added to each sample for chemical shift calibration and quantitation.

Metabolite concentrations were determined by peak integration, normalized to the peak integral of the respective internal standard (TSP for ^1^H and MDP for ^31^P MRS).

### Cell culture and drug treatment

A cell line of ERBB2/HER2-induced mammary carcinoma cells was established from a tumour mass excised from an MMTV-NEU-NT mouse [15,16]. The tumour cells were cultured in DMEM supplemented with 10% FCS, 100U/mL penicillin and 100 μg/mL streptomycin at 37°C in 5% CO_2_. Cell growth inhibition (96-hours) for these tumour cells, with seeding density of 1 × 10^3^ in 200 μL using 96 well plates, was measured by sulforhodamine B (SRB) assay to assess GI_50_[32]. Cells were treated with 17-AAG at pharmacologically active concentrations corresponding to 5 x GI_50_ (1.12 μM) for 24-hours at 37°C. The cells were collected by scraping, and viability was checked by the trypan blue exclusion assay [26]. The effect of treatment on viable cell number was monitored by counting the number of attached cells in the treated flask and comparing that with the number of attached cells in the control flask.

### Cell cycle analysis

Cell cycle analysis of attached control and treated cells was performed on cells (1 × 10^6^) fixed in 70% ethanol, treated with 100 μg/mL RNase A in citrate-buffered saline for 30 minutes at 37°C and stained with 4 μg/mL propidium iodide [33]. Samples were analysed on a BD LSR II (San Jose, CA, USA) on a “low” flow rate and excited using a 488 nm laser beam, fluorescent light collected at 610/20 nm on a linear scale for the cell cycle analysis. Concurrently data were collected at 660/20 nm on a log scale to assess samples for endoreduplication. The flow cytometry data were analyzed and quantified using the WinMDI and Cylchred softwares (University of Wales College of Medicine, Cardiff, UK).

### High resolution ^31^P-MRS of cultured cell extracts

5x10^7^ cells, approximately in log phase, were extracted from cell culture as previously described [34,35]. The freeze-dried extracts were resuspended in D_2_O with 10mM EDTA (pH 8.2).

^1^H-decoupled ^31^P-MRS spectra were acquired at room temperature on a 500 MHz Bruker spectrometer (Bruker Biospin, Coventry, UK) using a 30° flip angle, a 1s relaxation delay, spectral width of 100 ppm, and 32 K data points. Metabolite contents were determined by integration, normalized relative to the peak integral of an internal reference of MDP (60 μL, 1 mM), and corrected for signal intensity saturation and the number of cells extracted per sample.

### Western blot analysis

Standard Western blotting procedures were performed as described previously [36]. Briefly, cells were trypsinized, washed with PBS and lysed at 4°C in lysis buffer (Cell Signalling, Hertfordshire, UK) and protease inhibitor cocktail (Roche diagnostics, Mannheim, Germany). Protein concentrations were determined by the BIO-RAD assay method and bovine serum albumin as a standard. Antibody binding was identified with horseradish peroxidase-labeled secondary antibodies combined with enhanced chemiluminescence reagents (Amersham, Bucks, UK) and autoradiography.

Freeze-clamped tumours were lysed in 1ml of lysis buffer (0.1% Nonidet P-40, 50mM HEPES (pH 7.4), 250mM NaCl, 1mM phenylmethylsulfonyl fluoride, 10ug/ml aprotonin, 20uM leupeptin, 1mM dithiothreitol, 1mM EDTA, 1mM NaF, 10mM β-glycerophosphate and 0.1mM orthovanadate), protein concentration determined by the BCA protein assay (Thermo Scientific Pierce) and Western blotting procedures were followed as above.

Antibodies used were HSP72 (SPA 810; Stressgen, Canada), B-RAF (SC5284; Santa Cruz Biotechnology, Santa Cruz, CA, USA), C-RAF (SC133; Santa Cruz Biotechnology, Santa Cruz, CA, USA), CDK4 (SC260; Santa Cruz Biotechnology), p-NEU (2247; Cell Signaling Technology), NEU/HER2 (SC284; Santa Cruz Biotechnology), and also glyceraldehyde-3-phosphate dehydrogenase (GAPDH; MAB374; Chemicon, Hampshire, UK) as a loading control.

Images of the blots of solid tumor and cell extracts were scanned on a ImageScanner III (GE HealthCare) using Labscan software, and bands were quantified by densitometry using the ImageQuant TL software.

### Statistical analysis

The tumour cell data, solid tumour and tumour extract data are presented as the mean ± SEM, n = 3-6. For comparison of metabolite concentrations and tumour volumes, two-tailed unpaired t tests were used, and a p value of <0.05 was considered to be statistically significant.

## Results

### Dose-dependent effects of 17-AAG on tumour volume

The MMTV-NEU-NT mice developed multiple tumours associated with the mammary gland between 18-20 weeks of age. The tumour volumes at day 0, the day preceding the first dose of treatment, were 0.59 ± 0.08cm^3^ (n = 11), 0.67 ± 0.07cm^3^ (n = 11), 0.67 ± 0.08cm^3^ (n = 10), and 0.61 ± 0.12cm^3^ (n = 6) for the control 20, 40 and 80mg/kg cohorts respectively.

Tumours in the control mice (treated with vehicle only) had a doubling time of 12 ± 2 days (n = 6) and grew to 128 ± 6% of pre-treatment volume after 4 days, while the tumours treated with 3 daily doses of 17-AAG at 20mg/kg, 40mg/kg and 80mg/kg regressed to 88 ± 3 % (p < 0.003), 67 ± 6% (p < 0.003) and 42 ± 7% (p < 0.0005) respectively (compared with pre-treatment volume on day 0) by day 4. When the tumour volumes at day 4 post-treatment were compared to untreated tumour volumes at day 4, there were highly significant differences (p < 0.0002) at all doses (Table 1). There were significant differences in tumour volumes between the 40mg and 80mg/kg cohorts (p < 0.03) and between the 40mg and 20mg/kg cohort (p < 0.02). These results show that the NEU-NT tumours are very sensitive to treatment with 17-AAG and demonstrate dose-dependence of the therapeutic effect. The responsiveness is consistent with the results observed in a clinical study of trastuzumab-refractory HER2-positive metastatic breast cancer using 17-AAG in combination with trastuzumab [1,2].

**Table 1:** Effect of 17-AAG on tumour volume

	**Tumour volume (% of day 0) at day 4 (N)**	**P compared to day 0**	**Tumour volume (% of control) at day 4)**	**P compared to Control at day 4**
Control	128.4 ± 5.9 (11)	0.0007		
17-AAG (20mg/kg)	88.5 ± 2.9 (11)	0.003	68.9%	<0.0001
17-AAG (40mg/kg)	67.0 ± 3.6 (10)	<0.0001	52.2%	<0.0001
17-AAG (80mg/kg)	42.1 ± 7 (6)	0.0004	32.8%	<0.0001

Effect of 3 daily doses of 17-AAG (20mg/kg, 40mg/kg, 80mg/kg) or vehicle only (control) on tumour volume in MMTV-NEU-NT transgenic mice. Tumour volume expressed as a % of day 0 (column 2), p values: day 4 vs day 0. Tumour volume also expressed as a % of control cohort at day 4 (column 4), p values: treated day 4 vs control day 4. Mean tumour volume at day 0 of the 4 cohorts was 0.59 ± 0.08 cm^3^ (control), 0.67 ± 0.07 cm^3^ (20mg/kg), 0.67 ± 0.08 cm^3^ (40mg/kg) and 0.61 ± 0.12 cm^3^ (80mg/kg). Data are presented as the mean ± SEM.

**Figure 1:**
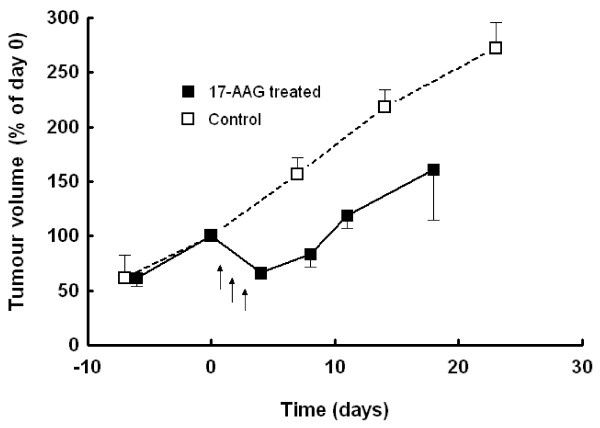
** Effect of 17-AAG on tumour volume of MMTV-NEU-NT tumours in transgenic mice: Mice were treated with 17-AAG (40mg/kg, days 1-3) with subsequent regrowth (■), vehicle treated controls (□).** Tumour volume is expressed as % of volume at day 0. Data are presented as mean ± SEM (n = 4).

In one cohort of mice (n = 4), where the animals were not sacrificed immediately after treatment (3 daily doses of 40 mg/kg 17-AAG), the tumours subsequently regrew at the original rate after the end of treatment (Figure 1).

### Effects of 17-AAG on molecular biomarkers in MMTV-NEU-NT tumours

**Figure 2:**
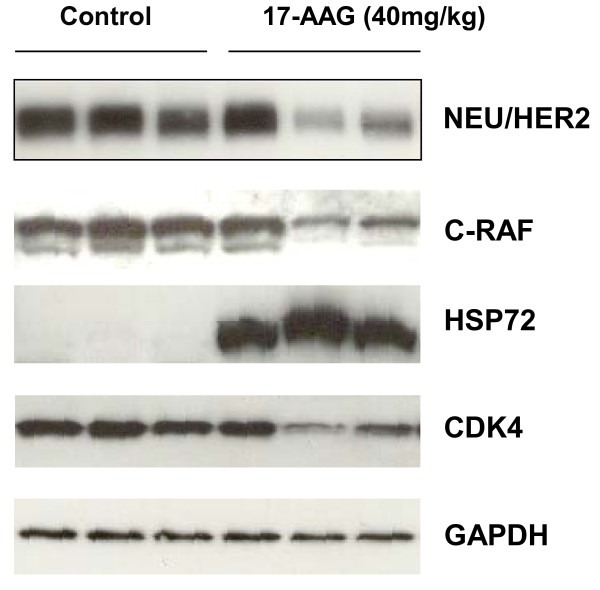
** Effect of 17-AAG on HSP90 biomarkers in MMTV-NEU-NT tumours : Western blots are shown for NEU/HER2, C-RAF, heat shock protein HSP72 and CDK4 expression in MMTV-NEU-NT tumours following treatment in mice with vehicle control (lanes 1-3), and 17-AAG, 40mg/kg, days 1-3 (lanes 4-6).** Tumours were excised 24 hours after the last dose. GAPDH was used as a loading control.

HSP72 induction is known to be a sensitive indicator of HSP90 inhibition and is used alongside client protein depletion as a validated biomarker signature of HSP90 inhibition [37]. Western blots for HSP72, NEU/HER2, C-RAF and CDK4 were carried out on tumours from a cohort of 3 control mice and 3 mice treated with 40mg/kg of 17-AAG for 3 days, where tumours were excised 24 hours after the last dose. The expected mechanism-based increase in HSP72 expression [37,38] was observed in all 3 treated tumours, and a decrease in the expression of NEU/HER2, C-RAF and CDK4 was seen in 2 out of 3 tumours (Figure 2). The intensity of each protein band was determined relative to GAPDH. The mean normalized band intensity of NEU/HER2 decreased from 3.64 ± 0.06 to 1.86 ± 0.37 (p = 0.009); C-RAF decreased from 3.01 ± 0.04 to 1.47 ± 0.29 (p = 0.006); HSP72 increased from zero to 3.12 ± 0.25 (p = 0.0002); and CDK4 decreased from 2.1 ± 0.07 to 1.22 ± 0.1, (p = 0.002). Extracts of tumours from the 20mg/kg cohort, taken 8hrs after the third dose, also showed consistent increases in HSP72 in all tumours and decreases in the expression of NEU/HER2 and phospho-NEU/HER2, C-RAF and CDK4, in 4 out of 6 tumours The mean band intensity, relative to GAPDH, of NEU/HER2 decreased from 1.09 ± 0.02 to 0.67 ± 0.14 (p = 0.03); phospho-NEU/HER2 decreased from 0.58 ± 0.11 to 0.12 ± 0.11 (p = 0.03); C-RAF decreased from 0.21 ± 0.05 to 0.04 ±0.02 (p = 0.04); CDK4 decreased from 0.23 ± 0.04 to 0.05 ± 0.03 (p = 0.02); and HSP72 increased from zero (undetectable) to 0.35 ± 0.1 (p = 0.02). Data are expressed as mean ± sem, n = 3 for control tumours and n = 6 for treated tumours. Taken together, these results show that 17-AAG caused a significant depletion of HSP90 client proteins, including NEU/HER2 in the MMTV-NEU-NT tumour model at 20mg/kg and 40mg/kg.

### Effects of 17-AAG on PC and PE levels in MMTV-NEU-NT tumours measured by high resolution ^1^H and ^31^P MRS in tumour extracts

**Figure 3:**
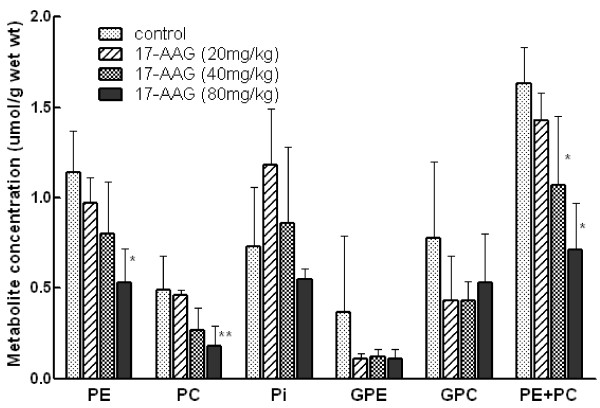
** Effect of 17-AAG on phospholipid related metabolites and Pi by**^**31**^**P MRS in extracts of MMTV-NEU-NT tumors: 3 daily doses of 17-AAG (20, 40, 80 mg/kg) or vehicle only (control) and tumours sampled at 24 hr post 3**^**rd**^**dose**. Data are expressed as mean ± SEM., n = 3 to 6, **p < 0.05, *p = 0.06 when compared to controls. Phosphoethanolamine (PE), phosphocholine (PC), inorganic phosphate (Pi), glycerophosphoethanolamine (GPE), glycerophosphocholine (GPC).

^31^P MRS of extracts allows better resolution of the signals than spectra acquired from *in vivo* tissue. Spectra of extracts of tumours from mice that had received 3 daily doses of 17-AAG were acquired, and individual phospholipid metabolites, i.e. PC, PE, GPC and GPE, were observed. Tumours from mice treated with 3 doses of 80mg/kg 17-AAG showed decreases in PE (p = 0.01), PC (p = 0.06) and [PE + PC] (p = 0.001). At the 40mg/kg dose the sum of [PE + PC] was significantly lower (Figure 3) when compared with vehicle-treated controls (p = 0.05). GPC and GPE levels were not significantly different at any of the 17-AAG dose levels when compared with vehicle-treated controls (Figure 3). ^1^H MRS of the extracts confirmed a significant decrease in PC in a cohort of tumours 24 hrs after treatment with 40mg/kg 17-AAG, but no other significant changes were observed (data not shown).

### Effects of 17-AAG on PME levels in MMTV-NEU-NT tumours *in vivo* measured by ^31^P MRS

**Figure 4:**
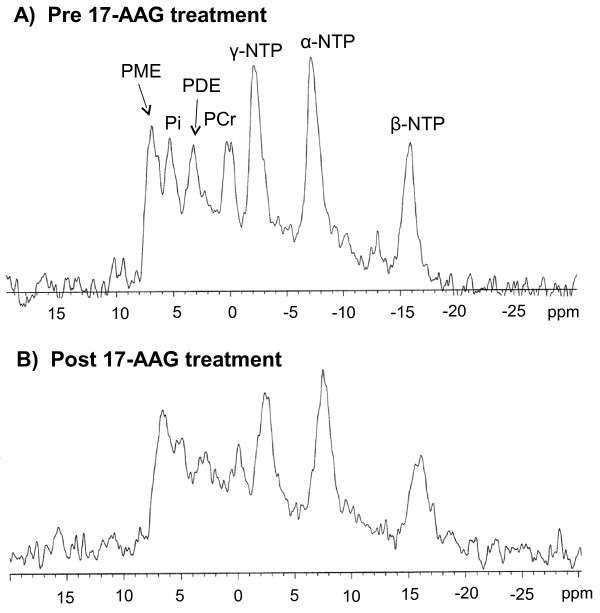
** Effect of 17-AAG on *in vivo***^**31**^**P MR spectra of MMTV-NEU-NT tumours in transgenic mice: 3 daily doses of 40mg/kg 17-AAG: A) pre-treatment with 17-AAG, B) 24 hrs after 3rd dose of 17-AAG.**

The metabolic response signature was also assessed as a potential non-invasive *in vivo* biomarker of HSP90 inhibition [26] pre and post 3 days of 40mg/kg 17-AAG treatment (see Figure 4). However, it was technically difficult to obtain spectra with a good signal to noise ratio at the 80mg/kg dose due to the marked tumour regression caused by the treatment (Table 1). This technical difficulty has been noted in other MRS experiments in drug-sensitive, rapidly regressing tumours [39]. No significant changes were noted in PME/Total P, PME/NTP, NTP/Pi ratios or intracellular pH in tumours pre- and post 17-AAG treatment at either the 20mg/kg or 40mg/kg dose (for 3 days) nor were there any significant changes in these ratios in the vehicle-treated controls.

### Effects of 17-AAG-treatment in cultured cells isolated from MMTV-NEU-NT tumours

The effects of 17-AAG on the MMTV-NEU-NT tumour extracts measured by ^31^P MRS were opposite to those seen previously in HT29 human colon tumours [26]; significant decreases (p ≤ 0.05) in the phospholipid metabolites PC and PE were observed in the MMTV-NEU-NT tumours, in contrast to significant increases in PC and PE (p ≤ 0.05) that had previously been seen in HT29 colon tumours. However, significant regression (p < 0.005) was seen at the 80, 40 and 20mg/kg doses in the MMTV-NEU-NT tumours compared to the tumour stasis that had previously been observed in the HT29 colon tumours, even at 80mg/kg. To investigate whether these differences were characteristic of the MMTV-NEU-NT tumours at the cellular level, we isolated and cultured cells from the MMTV-NEU-NT tumours. These cultured cells were then treated for 24 hours with 17-AAG at pharmacologically active concentrations corresponding to 5 x GI_50_ (1.12 μM) as defined using the 96 hours SRB assay. The number of 17-AAG treated cells per flask as a % of control showed a statistically significant reduction to 65.6 ± 6.1% (n = 6, P < 0.004), consistent with decreased proliferation.

**Figure 5:**
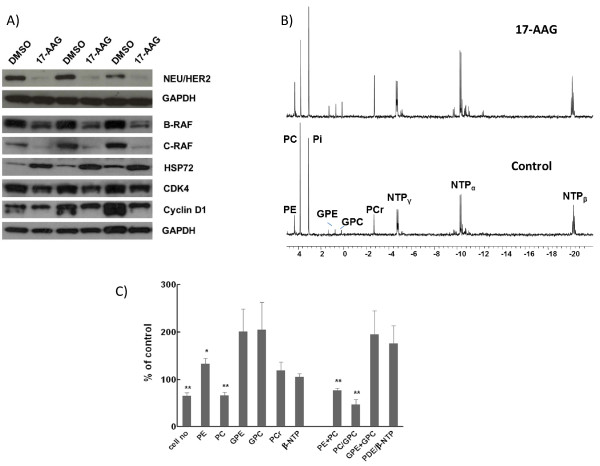
** Effect of 17-AAG on cultured cells isolated from a MMTV-NEU-NT solid tumour.** Cells treated for 24hr at 37°C with vehicle (control) or 1.12μM 17-AAG. **A)** Western blots of NEU/HER2, B-RAF, C-RAF, HSP72, CDK4 and cyclin D1 in vehicle treated (lanes 1,3,5) and 17-AAG treated (lanes 2,4,6) cells. Intensity of bands were quantitated relative to GAPDH, (n = 5). **B)**^31^P MR spectra of cell extracts from control cells (bottom), or cells following 17-AAG treatment (top). Peak assignments are: phosphoethanolamine (PE), phosphocholine (PC), inorganic phosphate (Pi), glycerophosphoethanolamine (GPE), glycerophosphocholine (GPC), phosphocreatine (PCr) and nucleotide triphosphate (α-, β-, γ-NTP). Results were confirmed in six separate experiments, and representative spectra are shown. **C)** Percentage changes (relative to control) in cell number, phosphoethanolamine (PE), phosphocholine (PC), glycerophosphoethanolamine (GPE), glycerophosphocholine (GPC), phosphocreatine (PCr) and nucleotide triphosphate (β-NTP), phosphomonoesters (PME = PE + PC), PC/GPC, phosphodiesters (PDE = GPE + GPC) and PDE/βNTP levels in MMTV-NEU-NT tumour cells following treatment with 17-AAG (1.12 μM) for 24h. Results are presented as the mean ± SEM (error bars) of six separate experiments. *p ≤ 0.05, **p < 0.01, two-tailed unpaired t test was used for all comparisons.

The MMTV-NEU-NT tumour cell line continued to overexpress NEU/HER2 in cell culture and this expression was largely abolished by 17-AAG treatment, as shown by western blotting (Figure 5A). The mean normalized band intensity of NEU/HER2 decreased from 0.34 ± 0.04 to 0.05 ± 0.005 (p < 0.001). There were also significant decreases in the normalized band intensity of C-RAF, from 0.53 ± 0.11 to 0.21 ± 0.07 (p < 0.05), and of cyclin D1, from 1.21 ± 0.1 to 0.85 ± 0.04 (p < 0.03). Normalized intensity changes of B-RAF (1.96 ± 0.16 to 1.84 ± 0.12) and CDK4 (0.88 ± 0.1 to 0.79 ± 0.05) were not significant. Treatment with 17-AAG also induced HSP72 expression, the normalized band intensity of which increased from 0.35 ± 0.04 to 0.97 ± 0.05 (p < 0.0001). Quantitation of band intensity is expressed relative to GAPDH, mean ± sem, n = 5 (Figure 5A). These results provide molecular evidence that HSP90 was inhibited in the 17-AAG-treated cultured mammary tumour cells, including marked depletion of NEU/HER2.

To further characterize the cellular effects of 17-AAG on cultured MMTV-NEU-NT mammary tumour cells, cell cycle distribution following 17-AAG treatment was determined by flow cytometry on the attached cell population. Exposure to 17-AAG induced a significant accumulation in the G1 phase population from 50.9 ± 1.4% to 72.2 ± 0.8% (n = 6, p < 0.0001) and a decrease in the S phase population from 40.3 ± 0.8% to 19.0 ± 0.9% (p < 0.0000001).

### Effects of 17-AAG on phospholipid metabolites in extracts of cultured cells isolated from MMTV-NEU-NT tumours measured by high resolution ^31^P MRS

^31^P-MR spectra of extracts of control and 17-AAG-treated (24hrs) mammary carcinoma cells cultured from MMTV-NEU-NT mouse tumours are shown in Figure 5B. Detailed analysis of the spectra demonstrated that 17-AAG treatment led to a statistically significant decrease in PME (76.8 ± 4.6%, p = 0.006). Of the signal contributing to the total PME, the largest proportion (83%) came from PC which also showed a significant decrease (66.7 ± 6.3%, P = 0.005). However, a small but significant increase was seen in the PE contribution (132.8 ± 11.3%, P < 0.05). GPE (200.8 ± 46.6%) and GPC (204.4 ± 57.2%) both showed increases but did not reach significance (p = 0.11 and 0.16 respectively) (Figure 5C). The ratio of PC/GPC also showed a significant decrease (46.7 ± 10.9%, p < 0.01).

## Discussion

The transgenic MMTV-NEU-NT mouse is a suitable and relevant model for ERBB2-driven human breast cancer [15]. It has been used in the present study to investigate effects of inhibition of the molecular chaperone HSP90, both because ERBB2 is one of the client proteins most sensitive to HSP90 inhibition and because of the clinical activity seen with 17-AAG in ERBB2-positive, trastuzumab-refractory breast cancer patients [1,2]. The results reported in this paper show that the MMTV-NEU-NT tumours are very sensitive to 17-AAG, confirming that NEU/HER2 is a prime target downstream of 17-AAG. Dose-dependent regression of the MMTV-NEU-NT tumours was seen at doses of 20, 40 and 80mg/kg (Table 1) whereas the drug has been found to be cytostatic in human tumour xenografts, including the HT29 colon tumour that we studied previously [26]. Tumour regrowth occurred soon after the treatment ceased (Figure 1). This suggests that when treatment is withdrawn, NEU/HER2 is able to recover its potent oncogenic activity. Most importantly, the regressions seen in the transgenic model recapitulated the regressions seen in the ERBB2-positive breast cancer patients.

The molecular effects of 20mg/kg or 40mg/kg 17-AAG on the transgenic MMTV-NEU-NT (Figure 2) tumours included the induction of the co-chaperone HSP72 and depletion of client proteins NEU/HER2, C-RAF and CDK4 (Figure 2). These results are consistent with the well-established molecular signature of HSP90 inhibitors [7,10,11,37,38,40]. Similar molecular changes, in addition to down-regulation of cyclin D1, were observed in isolated, cultured NEU/HER2 mammary cells following treatment with 17-AAG (Figure 5A).

No significant changes were seen *in vivo* by ^31^P MRS, possibly because the tumours regressed so rapidly that the signal/noise from the treated tumours was too low for effects to be quantifiable. Studies performed on tumour extracts at higher resolution *ex vivo,* however, did give statistically significant results. Increasing doses of 17-AAG caused a progressive fall in the mean concentrations of PE and PC measured in tumour extracts (Figure 3). Interestingly, these were only significantly different at the higher doses, when substantial tumour regression had taken place. Extracts of cells cultured from the tumours also showed a significant decrease in PC and total PME (PC + PE). However the PE component (17%) of the PME peak showed a significant increase when expressed as a % of control cells. PC, a cytosolic intermediate for the biosynthesis of phosphatidylcholine and a major phospholipid component of human cellular membranes, can be a useful indicator of tumour progression and therapeutic response [41]. In several tumour types that have regressed after treatment, a decrease in PC has been seen [41], similar to the changes observed in the extracts of both MMTV-NEU-NT tumours *in vivo* and cells cultured from these tumours, in this study. However in other cell and tumour types, an increase has been seen (reviewed in [25-27]) and more recently, PC was unchanged in a melanoma cell line [42] when treated with 17-AAG. The MRS metabolic response signature in solid tumour extracts showed some differences to that observed in the cultured mammary tumour cells - i.e. a decrease in PE in solid tumours in contrast to an increase in PE in the cultured cells. However, the tumours *in vivo,* unlike the isolated cultured tumour cells, would of course contain many other cells including stromal cells and macrophages, which may well confound the metabolic signature. Although no gross histological differences were seen between the treated and untreated tumours, it is possible that factors associated with regression (which also made it technically difficult to obtain good MR spectra *in vivo*), contributed significantly to the metabolic signature measured by MRS.

The fall in PE and PC in response to 17-AAG, observed in solid tumour extracts in the present study (Figure 3) should be considered in the context of the tumour- and drug-dependent PE and PC responses that have been obtained with ^31^P MRS in anticancer drug studies on tumour models in rodents. A recent review [25] cites ^31^P MRS studies in which PE and/or PC increased in response to chemotherapy with five anticancer drugs: 5-fluorouracil, ifosfamide, LAQ824 and SAHA (histone deacetylase inhibitors) and 17-AAG. In contrast, ten drugs induced falls in PC and/or PE: cyclophosphamide, docetaxel, the choline kinase inhibitor MN58b, the vascular disrupting agent ZD6126, the HIF-1α inhibitor PX478, the PI3 kinase inhibitors LY294002 and wortmannin, the MAPK inhibitor U0126, and orlistate, a fatty acid synthase inhibitor. This suggests that the PE and PC responses are drug-dependent and perhaps also tumour model-dependent.

All the above studies, including our previous one on 17-AAG [26] were performed on subcutaneously implanted tumour models and/or on isolated cells. To our knowledge, the present study is the first in which ^31^P MRS has been used to monitor the effect of an HSP90 inhibitor on spontaneous transgenic mouse tumours that arose in their true tissue of origin, in this case the mammary gland. Spontaneous tumours in genetically-engineered animals have a number of potential advantages over subcutaneously implanted tumours for testing anticancer drugs [43]. In particular, their histology and vasculature in many cases more closely resemble those of tumours in patients [44]. Since MR spectra are derived from both the tumour cells and the host stroma, that aspect of the model is important; indeed the metabolic state of the tumour cells themselves is strongly influenced by factors such as blood supply, which is often less adequate in implanted tumours than in spontaneous tumours [45], and by the host inflammatory response.

The ^31^P MRS response observed in human tumours responding to chemotherapy *in vivo* in the clinic is often a decrease in PE and PC (usually observed as a combined PME peak). Negendank [41] performed a meta-analysis on data from human tumours treated with chemotherapy from eight studies and found that 26/34 tumours showed a decreased PME peak, with 8 unchanged, whereas 4/7 non-responders showed increased PME peaks with 3 unchanged [41]. It is interesting, therefore, that the spontaneous NEU-NT/HER2 overexpressing mammary tumours studied here also showed decreased PE and PC in response to 17-AAG. However, in view of the broad spectrum and apparent drug-specificity of the PE and PC results reported from previous studies with implanted tumours, it will be necessary to wait for further results from other anticancer drug studies on this tumour model with respect to more detailed interpretation of the relationship between MRS signature and treatment response.

## Conclusions

In this study we have determined the effects of 17-AAG in a transgenic mouse tumour model that is driven by and addicted to the activated form of the NEU/HER2 oncogene. It therefore represents a good model of ERBB2-amplified breast cancer in the clinic. The ERBB2 oncoprotein is highly dependent on HSP90 for its activity and stability. Importantly, we show for the first time that the transgenic mouse mammary tumours driven by NEU/HER2 show significant regressions in response to 17-AAG treatment. Importantly, this recapitulates the regressions also seen in ERBB2-positive breast cancer patients. Regressions were associated with a decrease in NEU/HER2 oncoprotein expression. The metabolic signature obtained by ^31^P MRS showed a decrease in choline phospholipid. This finding was essentially similar between solid mammary tumours treated *in vivo* and the isolated tumour cells treated in culture. The decrease in choline phospholipids is consistent with a reduced malignant potential and suggests a normalisation of the choline phospholipid metabolism. The MRS changes observed in the choline phospholipid metabolites are consistent with inhibition and depletion of oncogenic signal transduction proteins, including NEU/HER2, and with decreased cell proliferation and tumour growth inhibition. Hence the observed MRS signature provides a basis for the potential use of MRS as a preclinical and clinical biomarker tool for investigating the antitumour activity of HSP90 inhibitors, particularly in the context of NEU/HER2/ERBB2-driven breast cancer.

## Competing interest

The Institute of Cancer Research has a commercial interest in HSP90 inhibitors. PW, Y-L C, NMSA, SYS, MOL and UB are employees of The Institute of Cancer Research which has a rewards to inventors scheme. Work on the discovery of HSP90 inhibitors in PW’s group was funded by Vernalis and HSP90 inhibitors were licensed to Vernalis and Novartis.

## Authors’ contributions

LR performed the in vivo experiments, analysed the data, coordinated the study and co-drafted the manuscript; YLC performed the MRS of the tissue extracts; NS and LEJ acquired all the isolated cell data; SS and UB did western blots on the tissue extracts; ML provided useful discussion; MS and JRG participated in the original conception of the study, in the interpretation of the results, and co-drafted the manuscript, PW participated in the original conception of the study, supervised it and co-drafted the manuscript. All authors read and approved the final manuscript.
